# Prediction of cognitive decline in Parkinson’s disease (PD) patients with electroencephalography (EEG) connectivity characterized by time-between-phase-crossing (TBPC)

**DOI:** 10.1038/s41598-023-32345-6

**Published:** 2023-03-29

**Authors:** Ute Gschwandtner, Guy Bogaarts, Volker Roth, Peter Fuhr

**Affiliations:** 1 Department of Mathematics and Computer Science, University of Basel, Basel, Switzerland; 2 Departments of Neurology and of Clinical Research, University Hospital of Basel, Basel, Switzerland

**Keywords:** Cognitive ageing, Neuronal physiology, Neurodegenerative diseases, Parkinson's disease

## Abstract

The aim of the study is to identify the dynamic change pattern of EEG to predict cognitive decline in patients with Parkinson’s disease. Here we demonstrate that the quantification of synchrony-pattern changes across the scalp, measured using electroencephalography (EEG), offers an alternative approach of observing an individual’s functional brain organization. This method, called “Time-Between-Phase-Crossing” (TBPC), is based on the same phenomenon as the phase-lag-index (PLI); it also considers intermittent changes in the signals of phase differences between pairs of EEG signals, but additionally analyzes dynamic connectivity *changes*. We used data from 75 non-demented Parkinson’s disease patients and 72 healthy controls, who were followed over a period of 3 years. Statistics were calculated using connectome-based modeling (CPM) and receiver operating characteristic (ROC). We show that TBPC profiles, via the use of intermittent changes in signals of analytic phase differences of pairs of EEG signals, can be used to predict cognitive decline in Parkinson’s disease (p < 0.05).

## Introduction

EEG brain activity correlates with cognitive changes in patients with neurodegenerative disorders^1–4^, and can even predict future development of dementia in patients with Parkinson’s disease (PD)^5^. While power spectra are intra-individually reliable^6^, they do not contain information on the synchrony of brain activity, which has been shown to be altered in dementia^2,7,8^. As the brain is a system with rapid changes, dynamic functional connectivity (DFC) bears promise to present a still more realistic and sensitive way for detection of cognitive decline. Alterations of DFC have been shown to be closely related to cognitive decline in patients with Parkinson’s disease; DFC was measured here by the frequency of change between two different states in resting state fMRI^9^. Comparable methods to quantify DFC with EEG data may be based on the strength of correlations between all possible functional connections as measured by the PLI. They can be based on averaged connections over time^15^.

Alternatively, the duration of the time difference (measured in ms) between two connections (defined by PLI) can be used. The latter method is called “Time Between Phase Crossings” (TBPC). This method is similar to one used in fMRI^10^. Each connectivity pair has an associated time lag, the sum of which per time unit is called TBPC coefficient and represents the dynamics of rapid electrical activity in the brain. TBPC is based on two connections.

A disadvantage of this method is, however, that for the use of high-density EEG recordings, a huge and impractical number of features will be the result. Therefore, it is important to use a particular subset of electrodes, depending on the specific question. Finally, the TBPC coefficient can be calculated for the five common EEG frequency bands (Delta, Theta, Alpha1, Alpha2 and Beta).

In this paper, we hypothesize that TBPC patterns differ between healthy control (HC) subjects and patients with Parkinson’s disease (PD) and further, that these differences can predict future cognitive decline in PD patients. Several studies have investigated relationships between longitudinal EEG (or Magnetoencephalography (MEG)) changes and longitudinal cognitive deterioration in PD^2,4^. Previous research indicates that changes in power and signal complexity as well as reduced functional connectivity in the θ-frequency band (Theta) are risk factors for the development of cognitive decline in PD^5,11^. Furthermore, another relevant requirement for the development of prognostic markers for cognitive decline in PD patients is the generalizability to newly diagnosed and mildly affected PD patients.

The aim of this study was to apply a prospective method (QEEG-connectivity) to detect neurophysiological risk factors for cognitive decline in patients with PD. We tested the hypothesis that P_PD_ scores discriminate between PD Patients and healthy controls, when measured by CPM (connectome-based predictive modeling).

## Methods

### Participants

**Figure 1:**
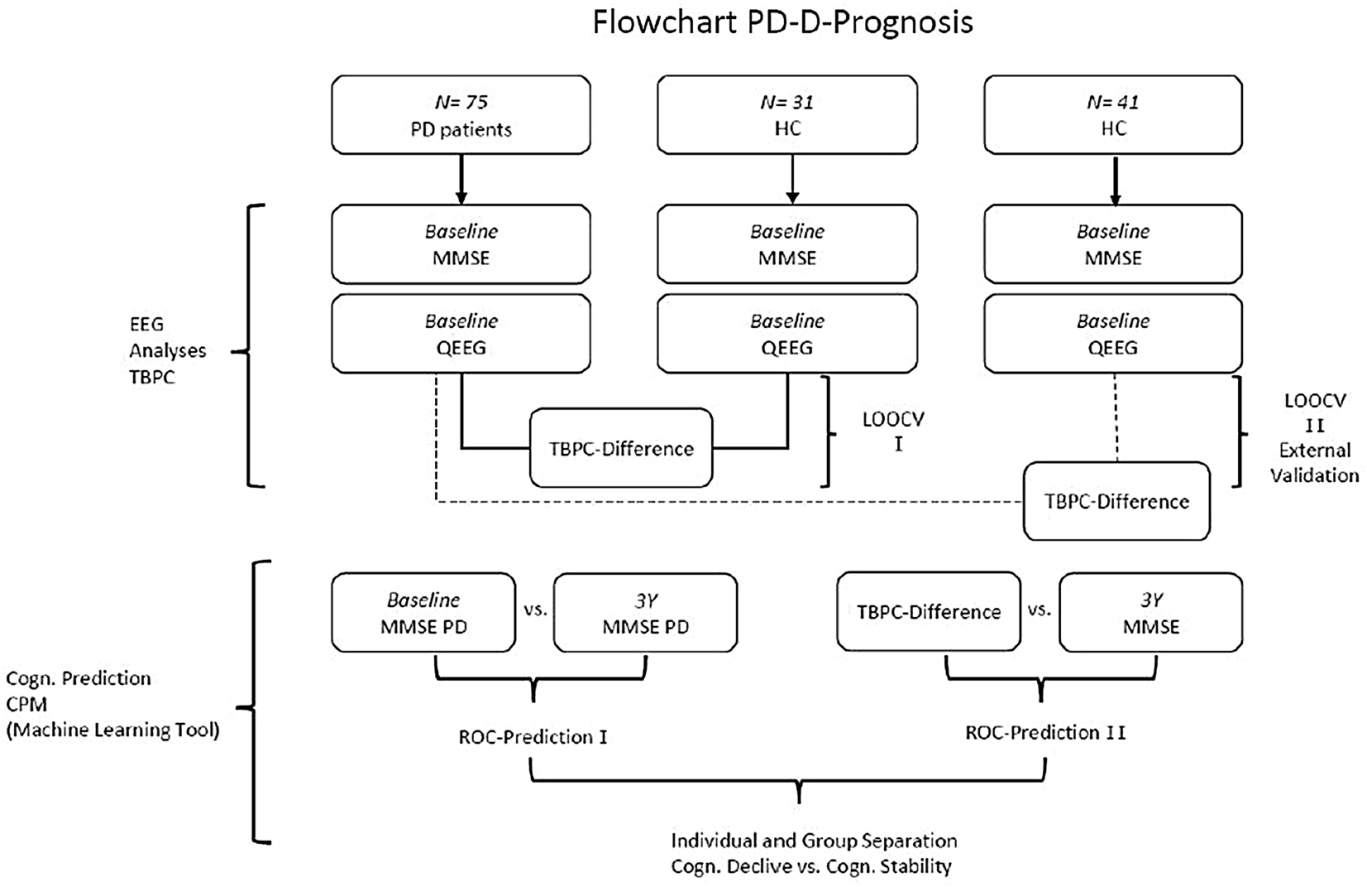
TBPC (time-between-phase-crossing) CPM (connectome-based modeling) MMSE (Mini Mental State Examination) QEEG (quantitative electroencephalography) LOOCV (leave-one-subject-out cross validation).

As part of a study funded by the Swiss National Science Foundation (SNSF, Nr. 159682) investigating cognitive decline in Parkinson’s disease, 79 non-demented PD patients were recruited (see Fig. 1). At baseline (BL), each individual underwent a 15-min, eyes-closed, resting-state EEG. Cognition was assessed using the Mini Mental State Examination (MMSE)^12^, converted to MoCA. We excluded four patients. One was considered to be a borderline dementia case, and another three had been diagnosed with early onset PD (aged under 50 years). For sample description of the patients at BL and after three years see Table 1. After 3 years, 54 patients underwent a follow up EEG as well as an MMSE assessment. All data was obtained in the “ON-medication state. As part of the study, 72 healthy control subjects (HC) were recruited and underwent the same EEG recording procedure. The cognition of the healthy controls was also assessed, which resulted in MMSE values of 29.5 points on average. To achieve groups of comparable sizes, the HC group consisted of two subgroups from earlier studies, which were not different and were examined similarly to the PD group. Then we related these results to the current and future cognitive status of the PD patients and HCs using independent t-tests with Bonferroni correction.

**Table 1:** Patients and healthy controls characteristics at baseline.

	PD patients			Healthy controls		
	Male	Female	P	Male	Female	p
N at baseline	50	25		37	35	
Age (years, median)	68 (50–82)	68 (56–80)	n.s.	70 (56–87)	69 (53–83)	n.s.
Education (years)	15.7 (10–20)	12.8 (9–20)	p < 0.001	12.5 (8–19)	12.5 (8–19)	n.s.
Disease duration (years)	5.3 (1–23)	6.3 (1–22)	n.s.	n.a.	n.a.	n.a.
MMSE at baseline	28.7 (24–30)	28.8 (26–30)	n.s.	29.5 (26–30)	29.5 (27–30)	n.s.
MMSE at 3 years	27.9 (22–30)	29 (26–30)	n.s.	29 (25–30)	28.5 (27–30)	n.s.

Age, education, disease duration in years (median and range), paired t-test.

All participants provided written, informed consent in accordance with a protocol approved by the local ethics committee (Ethikkommission Nordwest- und Zentralschweiz, Switzerland, (EKNZ)).

### EEG data acquisition & preprocessing

**Figure 2:**
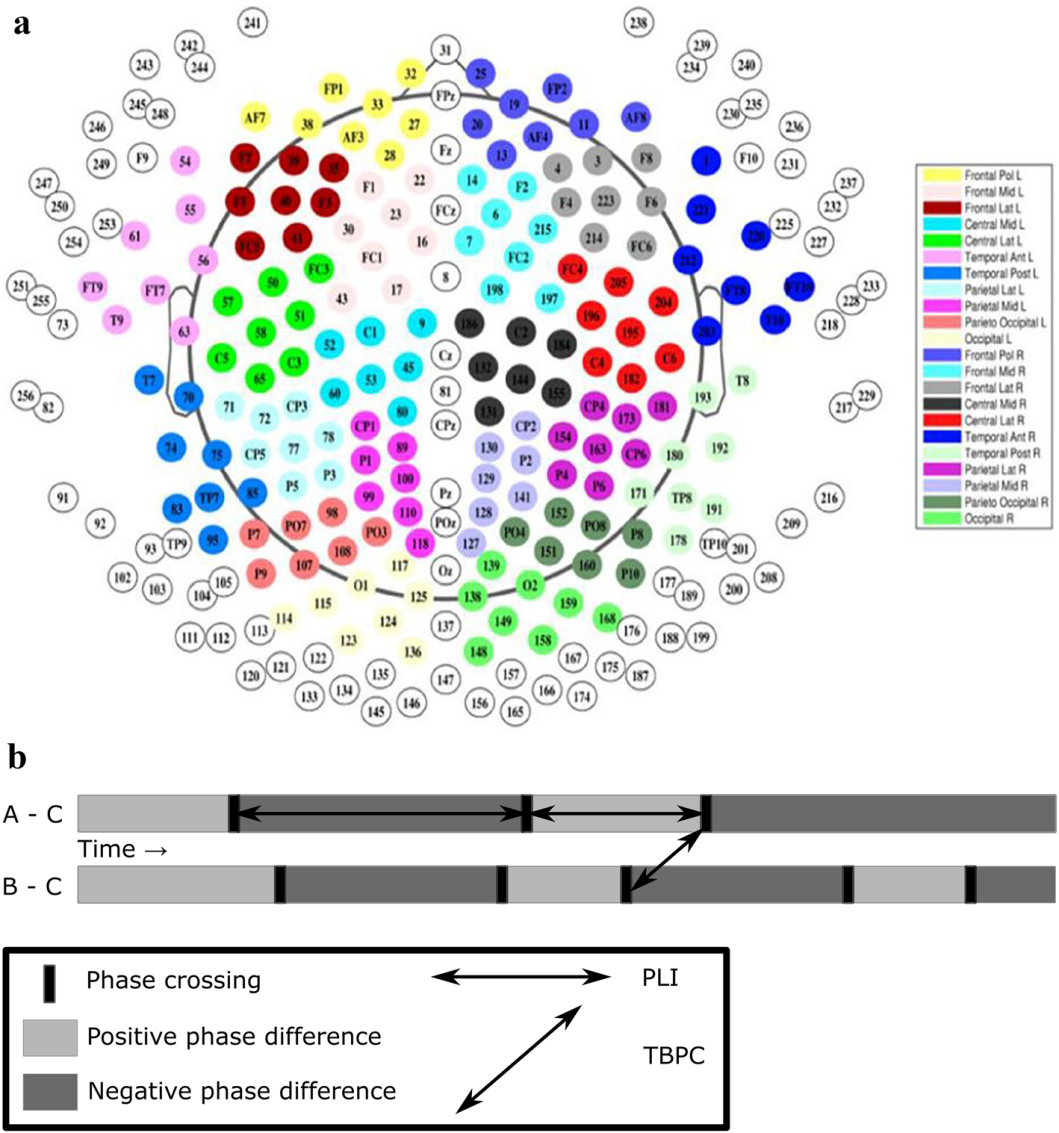
(**a**) 256-channel montage of EEG (only color-coded electrodes were analyzed). (**b**) Visual representation of the time course of phase differences between pairs of EEG signals. Each horizontal bar represents the phase differences between two EEG signals over time. Light and dark gray areas represent episodes with positive and negative phase differences, respectively. Vertical black bars mark transitions between positive and negative episodes (phase crossings).

EEG data was recorded using a 256-electrode Sensor Net® (Geodesics) (Fig. 2a). First, the correct Sensor Net size was determined by measuring the subjects head circumference. Next, the net was placed over the subject’s head, so that the central electrode (Cz), which served as the reference electrode, was located at the crossing of the midline and lateral line. Raw EEG signals were recorded with a sampling rate of 1000 Hz and filtered with a high-order, linear-phase, finite-impulse response filter (MATLAB: FIRLS (Finite Impulse Response Filter), 0.570 and 50 Hz notch, filter order: 4800). Only 213 out of 256 electrodes were used; electrodes located on the face and around the neck were excluded. After performing automated bad-channel detection^13^, the average of all ‘good’ channels was used to re-reference the EEG to a common average montage. Next, the EEG was band-pass filtered using a Butterworth filter, after which we calculated TBPC coefficients for each pair of electrodes. This was done separately for five commonly used frequency bands: δ: 1–4 Hz (Gamma), θ: 4–8 Hz (Theta), α1: 8–10 Hz (Alpha1), α2: 10–13 Hz (Alpha2), and β: 13–30 Hz (Beta). Given 213 electrodes, a TBPC profile consisting of 22,578 ((213 × 212)/2) TBPC coefficients was obtained for each frequency band. A short description of how TBPC coefficients are calculated will be given in the following.

In a first step, the PLI was calculated with the following formula:

$$\mathrm{PLI}=\frac{1}{T} \left|\sum_{t}^{T}sign\left[\mathrm{sin}(\Delta \phi \left(t\right))\right]\right|$$

where $(\Delta \phi \left(t\right)$ represents the analytic phase difference between two EEG signals at time point t. Starting with two EEG signals, one can visualize the results of each intermediate step of the PLI calculation. At this intermediate step of PLI calculation, the two EEG signals are converted to a vector of − 1 and + 1 values. At the next step, the sum over all time points is taken. This is analogue to calculating the difference in average duration of episodes with negative and positive phase differences (horizontal gray bars, Fig. 2b).

The method for calculating TBPC was conducted using baseline EEG signals. TBPC compares two connections measured by PLI, generated by three or four EEG signals. Four electrodes are usually required to generate two PLI connections. There is one exception to this rule: if both electrode pairs have one electrode in common, the TBPC is based on three electrodes only.

We restricted our analysis to a subset of TBPCs belonging to three electrodes, since one common electrode is located approximately halfway between the other two electrodes (Fig. 2b).

### Statistical classification and prediction procedures

To evaluate the ability of TBPC profiles to differentiate between HC subject and PD patients, and to predict MMSE scores for cognitive decline, we applied connectome-based predictive modeling (CPM)^14,15^ and Spearman’s rank correlation. A hallmark of CPM is that it takes high dimensional Functional Connectivity (FC) data and compresses them to only two variables: a positive and negative network strength. These two network strengths were subsequently used as independent variables in a regression model. Because FC values exist for five frequency bands, we have a total of ten independent variables. CPM model building consists of the following steps:

- Univariate testing of TBPC coefficients for significant differences or correlations
- Selecting features with a p-value below a given threshold
- Normalizing features to z-scores, based on the mean and standard deviation of the training data
- Averaging all selected TBPC-features into a positive (PD > HC) and negative (PD < HC) score, for each frequency band separately
- Fitting a regression model to the average scores and the outcome parameter (disease status or MMSE score)
- Testing the regression model
- Finally, to test the external cross validation of the model, we used leave-one-subject-out cross validation (LOOCV).

### Statements

All methods were carried out in accordance with all relevant guidelines and regulations and the experimental protocols were approved by the local ethics committee (Ethikkommission beider Basel, Switzerland, (EKNZ)). Informed consent was obtained from all participants prior to the start of the data collection.

## Results

### Overall cognition at baseline and after 3 years

At baseline, MMSE scores of the patients ranged from 24 to 30 points, with 68 out of 75 patients having a score of 28 points or higher (91%). After 3 years, MMSE scores of the patients ranged between 22 and 30 points of which 43 out of 54 had a score of 28 points or higher (83%).

An existing dementia diagnosis at baseline (MMSE < 24) was an exclusion criterion for this study. Information regarding depression (BDI), motor impairment (UPDRS III), and levodopa equivalent dosage (LED) were used as potential confounders (Table 2). There was no significant difference between UPDRS III, BDI (Table 2) and LED at baseline and at follow-up after 36 months.

**Table 2:** Data are median (interquartile range).

	BL	3-years FU	p
Beck’s Depression Inventory (BDI)	7 (4, 10.5)	7.8 (4, 10.75)	n.s.
Levodopa equivalent dosage (LED)	600 (350.0, 964.5)	585 (331.25, 1076.25)	n.s.
Motor impairment (UPDRS III)	15 (6.0, 21.5)	18.25 (12.25, 21.75)	n.s.

Quartile refer to 25th and to the 75th percentile.

*UPDRS-III* Unified Parkinson’s Disease Rating Scale (higher scores indicate lower functioning).

### Classification of PD patients and HC subjects

**Figure 3:**
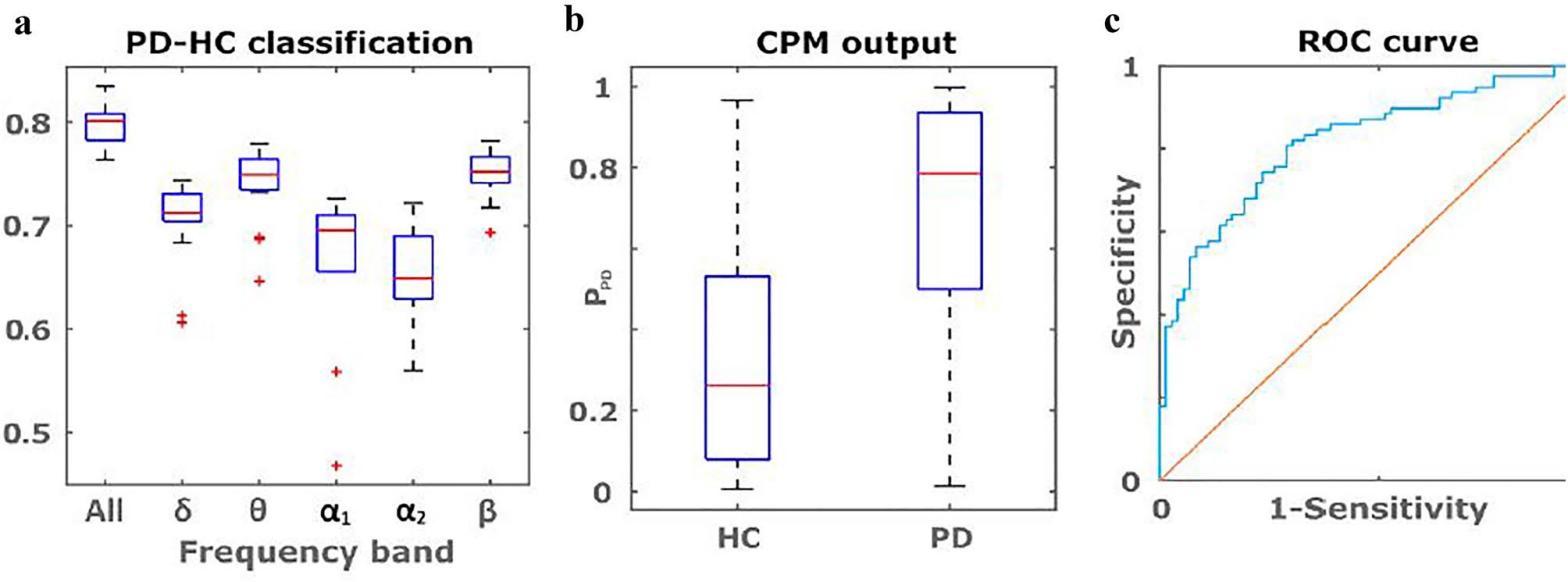
(**a–c**) PD-HC classification performance. (**a**) PD-HC classification using TBPC pro- files from all frequency bands together as well as for each frequency band separately. Box-plots indicate the distribution of AUC values obtained using different feature selection thresholds. (**b**) P_PD_ scores from the CPM classifier using TBPC profiles from all frequency bands together, averaged over all evaluated feature detection thresholds. (**c**) ROC value (0.8) is gives the probability to be P_PD_.

In line with the results described above, we observed that the differences between PD and HC were most pronounced in the θ- and β- frequency bands. Furthermore, single frequency band performance was lower compared to that of all the frequency bands together (Fig. 3a). First, we evaluated classification performance using TBPC profiles from all frequency bands. Depending on the feature selection threshold AUC values ranged between 0.65 and 0.80 (Fig. 3c). The CPM classifier outputs (PPD) represent the probabilities of an individual having PD or not, and are shown in Fig. 3b. The best performing CPM classifier consists of three parameters: negative network strength in the θ- frequency band and positive network strengths in the δ- and β-frequency bands. Classification performance was measured using the area under the receiver operation characteristics curve (AUC 0.80, Fig. 3c). Finally, we repeated the before mentioned analysis, but with using PLI instead of TBPC Overall, we observed a weaker and more variable performance for PLI, with AUC values ranging between 0.53 and 0.7 (data not shown).

### Current and predicted cognitive status of PD patients

**Figure 4:**
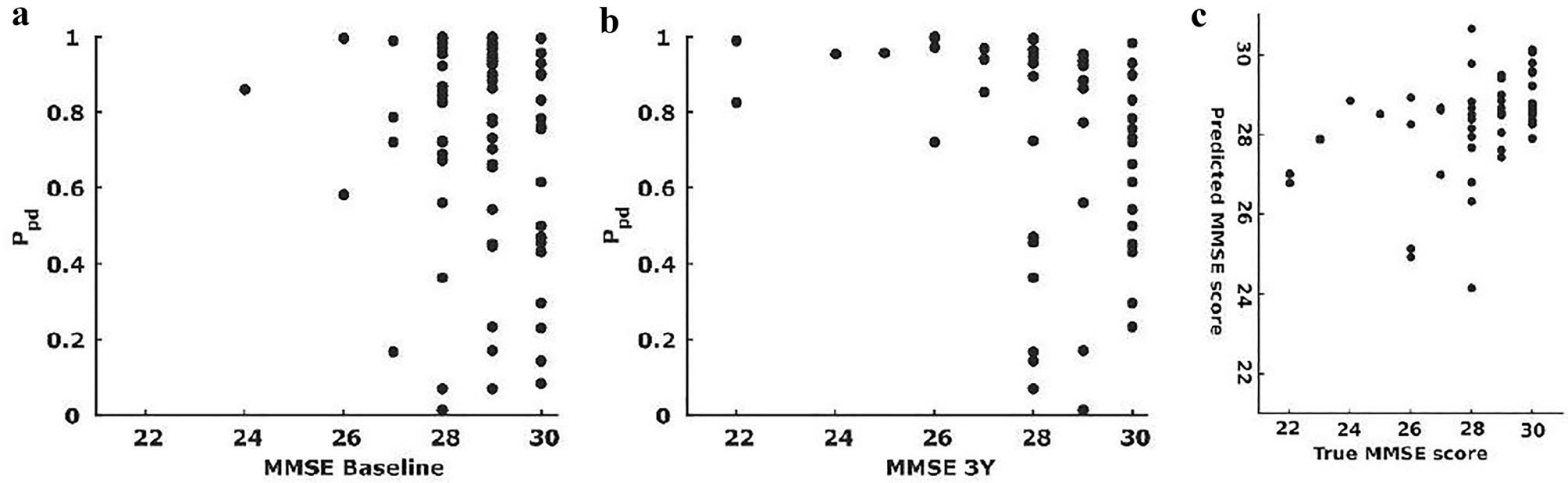
(**a**) MMSE baseline plotted against P_PD_ at baseline for 75 patients. Against baseline (r = −0.09), (**b**) MMSE3Y plotted against P_PD_ for 54 patients (r = −0.41), (**c**) predicted MMSE3Y plotted against true MMSE3Y (r = 0.33). Each dot represents a single patient.

As shown in Fig. 4a, we observed that PPD was not correlated with MMSE BL (r = −0.09, 95% CI: [−0.32 0.14], N = 75) whereas it was significantly correlated with MMSE3Y (r = −0.41, 95% CI: [−0.61 −0.16], N = 54, Fig. 4b,c).

For the prediction of cognitive decline, we assessed the statistical significance using Spearman’s rank correlation between actual and predicted MMSE 3Y scores. Using all frequency bands, the predicted and observed MMSE 3Y were significantly correlated (r = 0.33, 95% CI: [0.09 0.53]). When using single frequency bands, MMSE 3Y prediction performance was only significant for the θ- frequency band (r = 0.40, 95% CI: [0.15 0.60], Fig. 4c). The prediction analysis revealed that higher TBPC coefficients are associated with worse future cognition. Although the previous results showed that the TBPC profiles obtained from the β- frequency band contain information regarding MMSE 3Y, the signal-to-noise ratio of this pattern seems to be insufficient for enabling significant MMSE 3Y LOOCV results (r = 0.20, 95% CI: [−0.07 0.43]) (data not shown).

## Discussion

The dynamic QEEG measure TBPC used in this study contains prognostic information regarding the cognitive status of PD patients after 3 years—most notably in the θ-frequency band. Consequently, these results support both study hypotheses: (1) TBPC was able to differentiate HC subjects from PD patients, and (2) the length of TBPC in the θ-frequency band correlated with cognitive decline after 3 years in PD patients. These findings are also in line with previous research, demonstrating the relevance of the θ-frequency band in cognitive decline in PD^11,16^. Additionally to clinical perspectives, TBPC offers a view on one of the mechanisms at the base of cognitive decline, consisting in increased rigidity of brain activity.

Moreover, earlier quantitative analyses of electrical brain activity, employing a variety of methods, established the significance of EEG for measuring cognition in neurodegenerative disorders^4,5,17^. These studies often comprised of groups of patients with Parkinson’s disease, as well as with Alzheimer’s disease. Stam et. al.^1^ created a network analysis approach, which documented several hubs of connectivity decaying in parallel to cognitive decline, likely due to a period of functional overload. The microstate-analysis by Van de Ville et al.^18^ was one of the first developments of dynamic changes measured by QEEG in several brain disorders. A different approach was the estimation of information content using entropy measures employed in a study by Keller et al.^19,20^.

Apart from conflicting results regarding power analysis in the field of connectivity, one of the primary benefits of the PLI is its robustness against volume conduction. The proposed TBPC approach focuses on the dynamic aspects of brain communication, thus benefitting from the strengths of fMRI measures, while yielding information at a higher temporal resolution. Additionally, a high-density EEG system diminishes possible instability of the reference when re-referencing to the average of all electrodes.

While not yielding detailed information on the connectome, the TBPC opens a window on the dynamics of electrical brain activity and give some insight into the physiologic mechanisms of degenerative brain diseases^21^. Moreover, it might constitute a candidate prognostic biomarker for cognitive decline in patients with progressive brain disorders such as PD.

### Limitations and future research

One limitation of our investigation is that MMSE is the only measure of global cognition in this study, and not a detailed analysis of the complete array of cognitive capacities. While the current study presents results from an analysis of global EEG activity, the specific topology of EEG alterations in relation to cognitive decline might offer additional results, especially in connection with more differentiated neuropsychological profiles. Moreover, the PD as well as HC samples are relatively small, with only a slight cognitive decline, and the validity of the results is therefore limited by this fact and should be tested with independent cohorts.

## Conclusion

TBPC as a dynamic connectivity measure contains information about biomarkers for potential cognitive decline in PD patients. The detection of valid risk markers for cognitive decline and therefore the separation of groups of patients might be helpful in counseling the patients and can be used as inclusion criteria for several clinical trials dealing with future cognitive decline of patients with Parkinson’s disease.

## Data Availability

The data that support the findings of this study are available from P.F. upon reasonable request for reviewing purposes exclusively. The data are currently not available for the general research community because they are part of an ongoing clinical research project, of which important analyses have not yet been published.
